# Immediate and punitive impact of mechanosensory disturbance on olfactory behaviour of larval *Drosophila*

**DOI:** 10.1242/bio.20149183

**Published:** 2014-09-26

**Authors:** Timo Saumweber, Carmen Cano, Juliane Klessen, Katharina Eichler, Markus Fendt, Bertram Gerber

**Affiliations:** 1 Institut für Biologie, Universität Leipzig, Tierphysiologie, 04103 Leipzig, Germany; 2 Abteilung Genetik von Lernen und Gedächtnis, Leibniz Institut für Neurobiologie (LIN), 39118 Magdeburg, Germany; 3 Institut für Biologie, Universität Leipzig, Genetik, 04103 Leipzig, Germany; 4 Institut für Pharmakologie und Toxikologie, Medizinische Fakultät, Otto-von-Guericke-Universität Magdeburg, 39120 Magdeburg, Germany; 5 Center for Behavioral Brain Science (CBBS), 39016 Magdeburg, Germany; 6 Institut für Biologie, Otto von Guericke Universität Magdeburg, Verhaltensgenetik, 39106 Magdeburg, Germany; * Present address: Institut für Psychologie, Universität Bonn, 53111 Bonn, Germany.; ‡ Present address: Institut für Biologie, Universität Konstanz, 78457 Konstanz, Germany.

**Keywords:** *Drosophila*, Learning, Memory, Olfaction, Punishment, Mechanosensation

## Abstract

The ability to respond to and to learn about mechanosensory disturbance is widespread among animals. Using *Drosophila* larvae, we describe how the frequency of mechanosensory disturbance (‘buzz’) affects three aspects of behaviour: free locomotion, innate olfactory preference, and potency as a punishment. We report that (i) during 2–3 seconds after buzz onset the larvae slowed down and then turned, arguably to escape this situation; this was seen for buzz frequencies of 10, 100, and 1000 Hz, (ii) innate olfactory preference was reduced when tested in the presence of the buzz; this effect was strongest for the 100 Hz frequency, (iii) after odour-buzz associative training, we observed escape from the buzz-associated odour; this effect was apparent for 10 and 100, but not for 1000 Hz. We discuss the multiple behavioural effects of mechanosensation and stress that the immediate effects on locomotion and the impact as punishment differ in their frequency-dependence. Similar dissociations between immediate, reflexive behavioural effects and reinforcement potency were previously reported for sweet, salty and bitter tastants. It should be interesting to see how these features map onto the organization of sensory, ascending pathways.

## INTRODUCTION

*Drosophila* larvae have but 10,000 neurons, yet display a relatively rich behavioural repertoire (Vogelstein et al., 2014; for reviews, see Cobb, 1999; Diegelmann et al., 2013; Gerber and Stocker, 2007; Schleyer et al., 2013): they are not only able to feed, smell and taste, to sense visual, tactile and noxious stimuli, temperature and vibration, but also use these kinds of sensory information for learning. Larvae form associative memories between an odour and rewards such as fructose (Scherer et al., 2003) or low salt concentrations (Niewalda et al., 2008), whereas high salt concentrations or bitter substances as well as electric shocks can be used as punishment (Aceves-Piña and Quinn, 1979; El-Keredy et al., 2012; Niewalda et al., 2008). We focus on the behavioural impact of mechanical disturbance (‘buzzes’). In particular our experiments concern (i) the impact of buzzes on locomotion and on (ii) innate olfactory behaviour, as well as (iii) their potency as punishment (Eschbach et al., 2011).

Locomotion in larval *Drosophila* is studied mostly in Petri dish arenas covered with an agarose substrate. Their behaviour consists of runs, accomplished by peristaltic waves of muscular contraction that propagate from back to front (e.g. Gomez-Marin and Louis, 2014). Runs feature low-amplitude side movements (<20 degrees/s) of the first 1–3 segments, called head weathervaning. Weathervaning can support slightly curved runs and does not entail a break of the peristaltic wave (Gomez-Marin and Louis, 2014). Peristaltic runs can be interrupted to accommodate reorientation events. Upon such an interruption the larvae typically show more pronounced side movements of their head (∼60 degrees/s). Dependent on when the peristaltic wave is re-initiated during these movements, the body is pushed forward in this new orientation. The mechanosensory chordotonal organs and the brain hemispheres are apparently dispensable for these locomotor patterns, arguing they are produced by circuitry in the ventral nerve cord; however, brain and mechanosensory input are required for the integration of these locomotor patterns into adaptive, biologically meaningful behaviour (Berni et al., 2012; Caldwell et al., 2003; Ohyama et al., 2013; Wu et al., 2011).

**Fig. 1.**
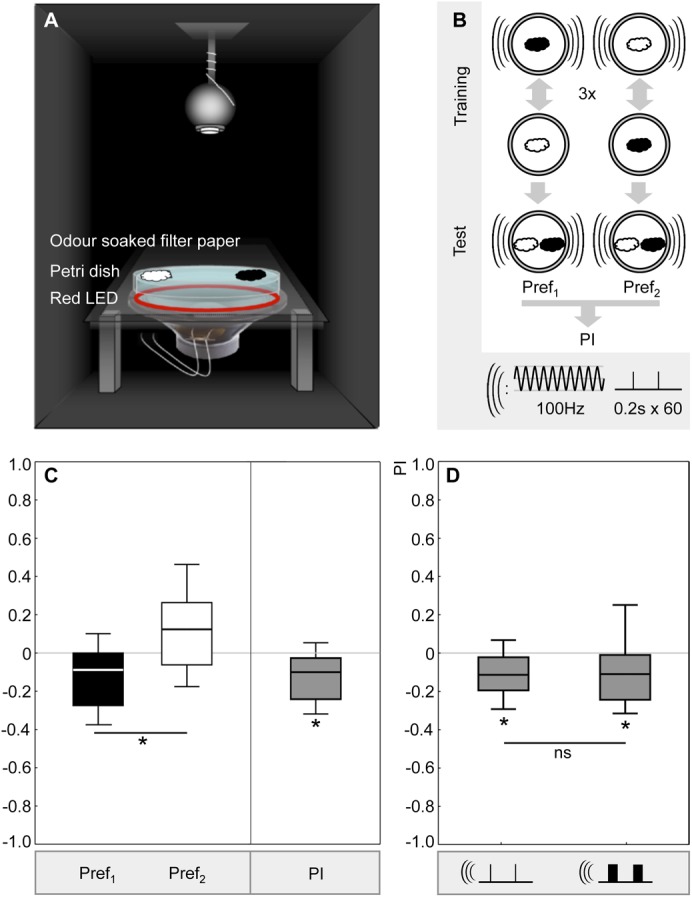
Buzz as punishment. (A) Experimental set-up: In a sound- and light-isolated box, a Petri dish was placed on top of a speaker delivering vibration as a mechanosensory punishment (‘buzz’). The dish was illuminated with a ring of red LEDs. Odours, represented as clouds, were applied using odour-soaked filter papers fixed to the lid of the dish. Larvae were allowed to crawl over the dish while one odour was presented together with the buzz, alternated with another odour being presented without the buzz (not shown). During testing a choice situation was created by placing different odours on either side, as shown. Larval behaviour was recorded with a digital camera mounted above the Petri dish. (B) Experimental design: during training, larvae either received a first trial with buzz punishment during the presentation of *n*-amyl acetate (filled cloud) and a second trial with the presentation of 1-octanol (open cloud) without the buzz. In a second group, larvae were trained reciprocally. These training cycles were repeated two more times. During the test, the larvae faced a choice between both odours. Preference scores were calculated on the basis of the number of larvae on either side; then, the associative performance (PI) was determined as the difference in preferences between the reciprocally trained groups, such that negative PIs indicate aversive memory. Please note that during testing the buzz was presented (see Introduction for rationale). The sequence of trial types was varied across repetitions of the experiment, making sure that in half of the cases *n*-amyl acetate or 1-octanol were first within training cycles, counterbalanced with buzz presentation during the first or second trial. During a punished trial, a 100 Hz buzz was presented every 5th second for a duration of 0.2 s, unless specified otherwise. (C,D) Results: to the left of panel C, odour preferences from the reciprocally trained groups are shown. Pref_1_ indicates the preference for *n*-amyl acetate when *n*-amyl acetate had been punished whereas Pref_2_ indicates the preference for *n*-amyl acetate for the reciprocally trained group in which 1-octanol had been punished. Data are presented as box-whisker plots, such that the middle line represents the median, the box the lower and the upper quartile, and the whiskers the 90 and 10 percentiles. * indicates a MWU-test of P<0.05; U = 506; N = 50, 50. To the right of panel C, the associative performance indices are presented; in this case * indicates significance from zero, that is an OSS-test of P<0.05; N = 50. (D) Increasing buzz duration from 0.2 s (left) to 2.0 s (right) did not affect associative performance scores (ns: MWU-test of P = 0.65; U = 643; N = 49, 29; * OSS-tests of P<0.05/2; sample sizes as above).

Interestingly in the current context, the presentation of a buzz can both interrupt peristaltic running (Eschbach et al., 2011; Ohyama et al., 2013; Zhang et al., 2013) and serve as punishment in an associative olfactory learning experiment (Eschbach et al., 2011) (both these effects may under natural conditions help larvae to avoid predatory wasps (Zhang et al., 2013)). In these experiments an odour A is presented with a buzz, but another odour X is presented without a buzz. After such training, the preference between both odours is tipped to the disadvantage of the previously punished odour (Fig. 1). In accordance with what has been found for other types of aversive olfactory learning in the larva (Apostolopoulou et al., 2014a; Apostolopoulou et al., 2014b; El-Keredy et al., 2012; Gerber and Hendel, 2006; Niewalda et al., 2008; Schleyer et al., 2011; Schroll et al., 2006; Selcho et al., 2009), such learned behaviour can best be understood as an escape strategy. Consider that you will not run out of a movie theatre upon seeing the emergency exit sign, but only when there **is** an emergency to escape from. Likewise, the larvae do not show conditioned escape during the test unless the punishment is present and escape indeed is warranted (for buzz as punishment (Eschbach et al., 2011)). In other words, the smell of the previously punished odour does not itself **trigger** escape, but gives **direction** to an escape which is otherwise triggered – namely by the buzz.

The current study aims to further our understanding of the behavioural impact of buzz-mechanosensation in larval *Drosophila*. We parametrically describe the potency of buzzes of various frequency (10, 100, 1000 Hz) as punishment, as well as their impact on free locomotion and olfactory preference behaviour.

## RESULTS

### Buzz as punishment

*Drosophila* larvae were trained such that one odour, namely either *n*-amyl acetate or 1-octanol (AM, OCT), was associated with a buzz as punishment (–). Then, the larvae were offered a choice between AM and OCT (Fig. 1A,B). Preference scores as displayed in Fig. 1C (left) were defined such that positive scores indicate a choice of AM while negative scores indicate a choice of OCT. We used ‘standard’ 0.2 s-duration buzzes at a frequency of 100 Hz (Eschbach et al., 2011). Preference scores were shifted towards OCT after AM–/OCT training as compared to AM/OCT– training (Fig. 1C, left). Correspondingly, the associative performance index, which measures the difference in preference, was significantly negative (Fig. 1C, right). Increasing buzz duration by a factor of ten, i.e. from 0.2 s to 2 s, did not increase this associative effect (Fig. 1D; for the underlying preference scores, see supplementary material Fig. S1), suggesting that the punitive effect of the buzz might be largely exerted by its onset (Zhang et al., 2013).

**Fig. 2.**
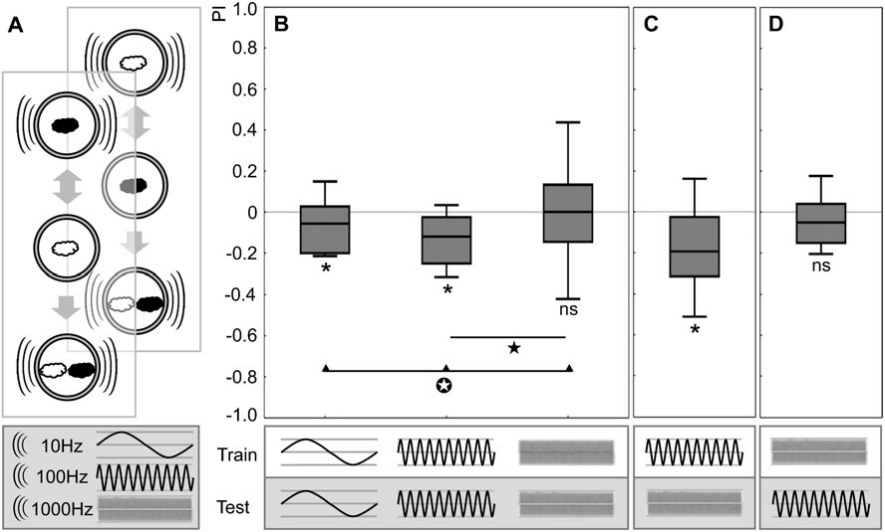
Buzz as punishment: frequency-dependence. (A) Sketch of the experimental design using buzz frequencies of 10 Hz, 100 Hz and 1000 Hz. Presenting the buzz during the test is required, because conditioned avoidance is not behaviourally expressed if there is no ‘reason’ to escape (see Introduction for rationale). (B) Associative performance indices when using buzzes at the indicated frequencies. Associative performance is observed for 10 Hz and 100 Hz, but not for 1000 Hz buzzes. * and ns refer to P<0.05/3 and P>0.05/3 (OSS-tests), respectively; 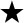 refers to P<0.05/2 (MWU-test, P<0.05/2; U = 327.0), 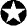 refers to P<0.05 (KW-test: P<0.05; H = 7.71; df = 2). From left to right, sample size is N = 32, 38, 28. (C,D) Associative performance indices when using buzzes differing in frequency between training and test. Odour-buzz memory, if established using a 100 Hz buzz, can be behaviourally expressed at 1000 Hz (C). In turn, 1000 Hz buzzes cannot function as punishment (D). * and ns indicate P<0.05 and P>0.05, respectively (OSS-tests) (N = 50, 43).

Next, we asked whether the frequency of buzz punishment has an influence on associative scores (Fig. 2B; supplementary material Fig. S2B). Buzzes of 10 Hz and 100 Hz support significantly negative associative performance indices, whereas 1000 Hz buzzes did not. A relatively low frequency of 10 Hz supported the same level of associative effect as the standard 100 Hz buzz, while the scores using 1000 Hz buzzes were less relative to the 100 Hz buzz.

We were surprised to observe that the 1000 Hz buzz did not support a punitive effect. As mentioned in the Introduction, both for bad-taste and for the buzz as punishment, learned behaviour is part of an escape process and is expressed only in the presence of the punishment. Therefore the lack of associative effect of a 1000 Hz buzz may either be because no odour-buzz memory is established, or because the 1000 Hz buzz during testing does not allow the behavioural expression of an otherwise intact odour-buzz memory. Given that the standard buzz of 100 Hz **was** effective as punishment (middle plot in Fig. 2B), we trained larvae with such a standard 100 Hz buzz, but tested them in the presence of a 1000 Hz buzz. It turned out that associative scores were intact (Fig. 2C; supplementary material Fig. S2C). This argues that a 1000 Hz buzz is permissive for learned escape. In turn, as the standard 100 Hz buzz **was** also permissive for learned escape (middle plot in Fig. 2B), we trained larvae with a 1000 Hz buzz, but tested them in the presence of the standard 100 Hz buzz. In such an experiment, associative scores were zero (Fig. 2D; supplementary material Fig. S2D). This argues that a 1000 Hz buzz does not support the establishment of an aversive memory to begin with.

We conclude that buzzes of 10 as well as of 100 Hz are effective as punishment, whereas buzzes of 1000 Hz are not.

Of note, the fact that 100 Hz buzzes induce aversive memory for concomitantly presented odours means that these odours are effectively processed towards the larvae's ‘memory centre’ during training. Also, 100 Hz buzzes allow for the retrieval of such olfactory memories, arguing that also during testing odours can be effectively processed towards and from this ‘memory centre’. Thus, the associative aspects of odour processing remain intact in the presence of a 100 Hz buzz.

### Buzz as modulator of innate olfactory behaviour

**Fig. 3.**
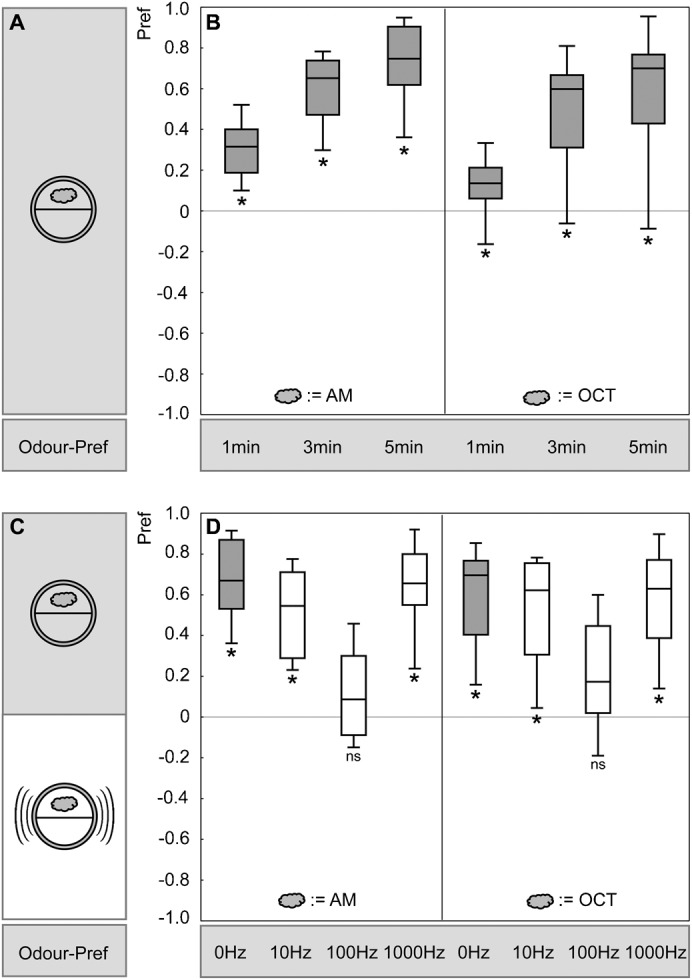
Buzz as modulator of olfactory preference. (A,B) Time course of olfactory preference. Larvae were offered the choice between an odour side and a blank side of a Petri dish. Preference was scored after 1 min, 3 min, and 5 min, using either *n*-amyl acetate (AM) or 1-octanol (OCT) as odours. For both odours, choice behaviour reached a steady state after 5 min; therefore these 5-min scores are displayed in panels C and D. * indicates significance from zero (OSS-tests, P<0.05/3) (N = 24 for AM and N = 23 for OCT preferences). (C,D) Modulation of innate olfactory preference behaviour by the buzz. Odour preferences, for AM or for OCT, are displayed, either assayed without the buzz, or assayed in the presence of the buzz at the indicated frequency. Buzzes of 100 Hz abolish innate olfactory behaviour. * and ns refer to P<0.05/4 and P>0.05/4, respectively (OSS-tests) (N = 24, 19, 19, 18; N = 23, 19, 18, 18).

We offered the larvae a choice between an odour side (either AM or OCT) *versus* a blank side of a Petri dish and recorded their preference – and did so either in the presence or in the absence of a buzz (Fig. 3A,C). Given that it takes 3–5 min for the larvae to distribute themselves between both sides of the Petri dish (Fig. 3B), we chose to focus on the data at 5 min. This was done for either 10, 100, or 1000 Hz buzzes. In the presence of 10 Hz and 1000 Hz buzzes the larvae behaved the same as larvae tested without a buzz; to our surprise, however, in the presence of 100 Hz buzzes innate odour preference was strongly decreased, for both the odours employed (Fig. 3D).

We conclude that 100 Hz buzzes, but not 10 or 1000 Hz buzzes, strongly modulate innate olfactory behaviour – while, as mentioned above, associative aspects of olfactory processing remain unaffected in the presence of a 100 Hz buzz.

### Buzz as modulator of locomotion

**Fig. 4.**
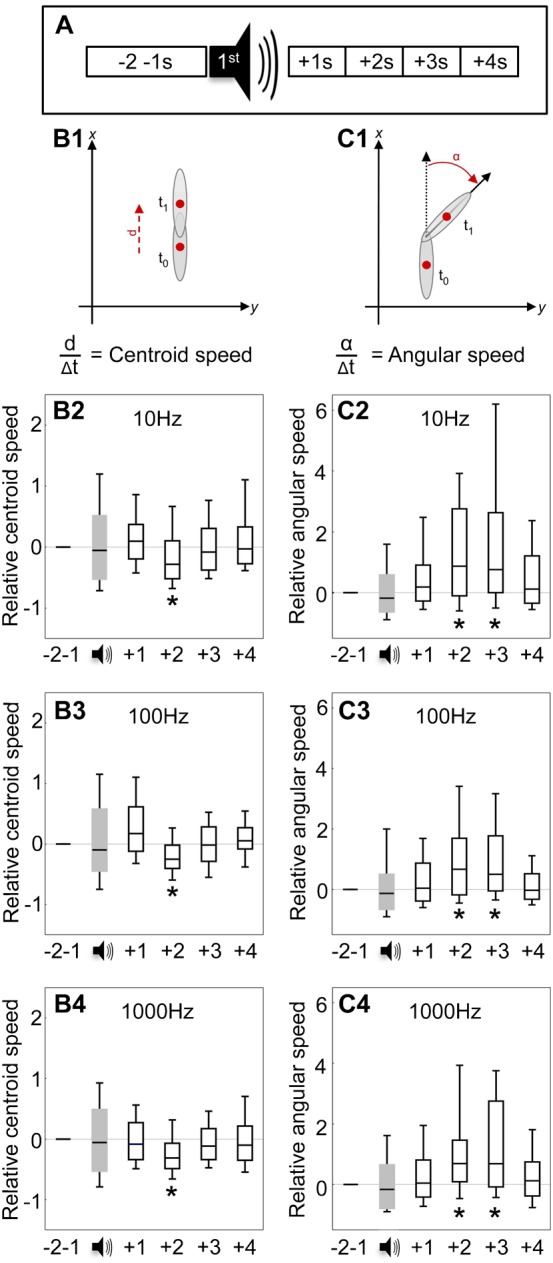
Buzz as modulator of locomotion. Larval locomotion was assayed 2 s before, during, and 4 s after buzz onset (A). Analysed parameters were centroid speed (B) and angular speed (C), displayed for the four 1-s time windows normalized to the 2 s before buzz onset, using buzzes of the indicated frequency. At all buzz frequencies, larvae slow down (@ 2 s), and turn (@ 2–3 s). * indicates P<0.05/5 (OSS-tests) (N = 88, 82, 84 for 10, 100 and 1000 Hz, respectively).

We monitored locomotion of individual larvae and quantified their innate behaviour with respect to buzzes of 10, 100, or 1000 Hz. We present speed and turning propensity upon the very first (Fig. 4), the 10th (supplementary material Fig. S3) and the 60th buzz within a 5 min period (supplementary material Fig. S4). We normalized data to the 2 s before the onset of the respective buzz as baseline. In keeping with Eschbach et al. (Eschbach et al., 2011), the larvae ‘startled’, that is they briefly slowed down and then turned in response to a 100 Hz buzz (Fig. 4B3,C3). The speed dropped below baseline at second 2, yet returned to baseline while turning was still in progress, until at second 3 to 4 after the buzz the new direction was assumed. The same qualitative pattern of results was found for 10 Hz and 1000 Hz buzzes (Fig. 4B2,C2,B4,C4). These results were surprisingly stable over dozens of repetitions of the buzz (for the 10th and 60th buzz, see supplementary material Fig. S3, Fig. S4).

We conclude that innate behaviour towards buzzes is a rather repetition-stable behaviour consisting of sequential slowing-down and turning, and that this behaviour does not depend on the frequency of the buzzes, at least not across 10, 100, and 1000 Hz.

## DISCUSSION

We demonstrate that mechanical disturbances (buzzes) impact immediate behaviour and are effective as punishment – and that buzzes of different frequency differ in impact across the types of behaviour assayed: 10 Hz buzzes function as punishment, do not modulate innate olfactory behaviour, and induce startle. Buzzes of 100 Hz also serve as punishment, do reduce innate olfactory preference and elicit startle. Lastly, 1000 Hz buzzes cannot serve as punishment, do not modulate innate chemotaxis, and do make larvae startle. How can these differences in frequency-dependence be understood?

For sugars, salt, and quinine, mismatches have been reported between the dose-effect functions of immediate and reinforcing effects (El-Keredy et al., 2012; Niewalda et al., 2008; Russell et al., 2011; Schipanski et al., 2008). For example, figure 5 in El-Keredy et al. found that the suppressing effect of quinine on feeding is shifted by about one order of magnitude towards higher concentrations as compared to the punishing effect of quinine (El-Keredy et al., 2012). The authors suggested that different sensory neurons differing in dose-effect function and differentially hooked up to feeding behaviour versus reinforcement signalling are responsible for these effects. This was confirmed by Apostolopoulou et al.: ablating *Gr33a*-Gal4 positive gustatory sensory neurons reduces feeding-suppression by quinine, but leaves punishment processing unaffected (Apostolopoulou et al., 2014b).

A buzz interrupts peristaltic running and induces a brief hunch, followed by large-amplitude sideways movements of the head and ensuing peristaltic runs into a new direction (Bharadwaj et al., 2013; Ohyama et al., 2013; Wu et al., 2011; Zhang et al., 2013) (Fig. 4). This sequential pattern of behaviour is reminiscent of startle in mammals (supplementary material Fig. S5): upon a sudden and intense visual, tactile or acoustic stimulus, mammals interrupt current behaviour, close their eyes, flatten their ears, bend their spine and limbs and stiffen their neck (these behaviours are typically measured as ‘startle’). In a second phase, the eyes are widely opened, the ears pricked, and, while the spine and body parts remain bent, the head is rotated sideways (Landis and Hunt, 1939; Strauss, 1929; Gerber et al., 2014; Koch, 1999; Yeomans and Frankland, 1995). As in larvae, these behaviours seem to initially protect the subject, followed by attempts at threat localization, reorientation, and preparation for a fight or flight decision.

Regarding the neurogenetics of sensing mild mechanical disturbance like buzzes, the precise targeting of chordotonal neurons within the central nervous system is required (Wu et al., 2011). Further, these chordotonal neurons are necessary for modulating head casts, crawling and hunching with respect to vibration and gentle touch (Caldwell et al., 2003; Fushiki et al., 2013; Ohyama et al., 2013; Wu et al., 2011). Within these chordotonal neurons, the natural sounds of wasps and yellow jackets as well as pure tones of 500 Hz are sensed by NOMPC, NANCHUNG, and INACTIVE channels (Zhang et al., 2013). In terms of sufficiency, optogenetic activation of chordotonal neurons evokes aspects of startle behaviour (Ohyama et al., 2013). Thus, activity in the chordotonal neurons seems largely necessary and sufficient for larval startle behaviour. Extracellular recordings of chordotonal neurons and Ca^2+^ imaging fit these conclusions in showing an optimum function with a peak at about 500 Hz (Zhang et al., 2013). Stimuli with 10, 100 or 1000 Hz, as used in the current study and in Eschbach et al., would, according to Zhang et al., induce only very moderate activity (Eschbach et al., 2011; Zhang et al., 2013). When summed up across all chordotonal neurons across all body segments, however, even such moderate activity may be sufficient for startle (Fig. 4).

To summarize, the different frequency-dependencies of how buzzes affect locomotion, innate olfactory preference, and their potency as a punishment, parametrically dissociate these three types of behavioural effects. It should be fascinating to map these dissociations, which likewise have been found for the taste system, onto the emerging behaviour-connectome relationships of the larva (Cardona et al., 2010; Cardona et al., 2012).

## MATERIALS AND METHODS

### Larvae

Third-instar feeding stage larvae of the Canton S strain were used, raised on standard food in groups of about 200, under standard conditions (25°C, 60–70% relative humidity, 12/12 light/dark cycle).

### Set up and stimuli

The experimental setup follows Eschbach et al. and Eschbach (Eschbach et al., 2011; Eschbach, 2011) (Fig. 1A). It consists of a 50×50×75 cm wooden box covered on the inside by silencing foam. A speaker (MC GEE 201847, CON Elektronik, Greussenheim, Germany, impedance 8 Ω, diameter 16 cm, acoustic pressure: 89.2 dBW^−1^ power 150 W r.m.s.) was fixed at the bottom, such that a 145 mm diameter Petri dish (Sarstedt, Nuembrecht, Germany) could be placed on top. An opaque inner ring made of Perspex and an outer ring fitted with 30 LEDs (624 nm, Conrad Electronics, Hirschau, Germany) surrounded the Petri dish. The Petri dish was covered with a thin layer of agarose (1%; electrophoresis grade; Roth, Karlsruhe, Germany) on the eve of the experiment. The speaker was operated via a PC using a custom-written LabVIEW program. A camera (Logitech Webcam Pro 9000, frame rate 30 s^−1^, Logitech, Munich, Germany) could be fixed above the plate and connected to a PC for offline analysis.

1-octanol (OCT, CAS: 111-87-5, purity: 99%) and *n*-amyl acetate (AM, CAS: 628-63-7, purity 98%, diluted 1:50 in paraffin oil, CAS: 8012-95-1) (all Merck, Darmstadt, Germany) were used as odorants. Filter papers (7 mm^2^) were loaded with 10 µl of AM or OCT and fixed to the Petri dish cover (50 mm from both the midline and the rim).

### Buzz as punishment

Behaviour is compared between reciprocally trained larvae. One set of larvae experienced AM with punishment (–) and OCT without punishment (AM–/OCT), while the other larvae were trained reciprocally (OCT–/AM) (Fig. 1B). For the test, the relative preference between the two odours was measured, allowing us to calculate an associative performance index as the difference in preference between the reciprocally trained larvae. In the next section, we present our ‘standard’ protocol; parametric variations are mentioned in the course of the Results.

Two filter papers were fixed to the Petri dish lid, both loaded with 10 µl of the same odour (e.g. AM) and the lid was put on the Petri dish. Then, 50 larvae were collected from their rearing vials, washed and transferred to the Petri dish. For punishment, 60 disturbances were applied at a frequency of 100 Hz (‘buzzes’ in the following), each lasting 0.2 s and presented evenly spaced in time for 5 min. The larvae were then transferred to a fresh Petri dish and OCT was presented, without the buzz (AM–/OCT). This cycle was repeated two more times (in half of the cases larvae were punished during the 1st, 3rd and 5th trial, while otherwise they were punished in the 2nd, 4th and 6th trial).

For testing, larvae were transferred to a Petri dish equipped with AM on one side and OCT on the other. After 5 min, we counted the number of larvae in the middle (0.5 cm wide stripe), on the AM side and on the OCT side. A preference index is calculated as:
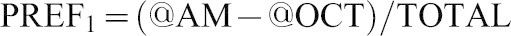
(1)Likewise, a preference index PREF_2_ was determined for larvae of the reciprocally trained group (AM/OCT–). The performance index PI was defined as the difference in preference between the reciprocally trained groups:
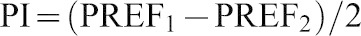
(2)Positive scores thus indicate appetitive memory, while negative scores indicate aversive memory, that is a punitive effect of the buzz. Testing was performed in the presence of the buzz (see Introduction for rationale).

### Buzz as modulator of olfactory preference

Two 7-mm^2^ filter papers were fixed to the Petri dish lid, one of which was loaded with odour (10 µl of either AM or OCT) while the other one was left blank. A group of 50 larvae was collected and transferred to the middle of an agarose-filled Petri dish. The Petri dish was then placed into the assay box described above. After 1, 3 and 5 min we determined the number of larvae on either the odour side or the blank side or the middle stripe, allowing us to calculate a preference score as:
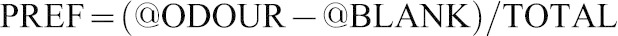
(3)This experiment was performed either as described, or in the presence of the buzz.

### Buzz as modulator of locomotion

We determined two key parameters of the behaviour towards the buzz, namely changes in speed and changes in turning propensity. Single larvae were observed for 5 min, moving over an agarose-filled Petri dish. During this time, buzzes of 0.2 s duration were presented, evenly spaced in time, and data were recorded for offline analyses. For the first buzz as well as for the 10th and the 60th buzz, we determined speed (mm/s; 1 voxel  =  0.33 mm) and turning propensity (°/s) (for details, see Eschbach et al., 2011; Eschbach, 2011). Baseline speed and turning propensity were determined for the 2 s before the buzz; data were then scored for the 1st, 2nd, 3rd and 4th second after onset of the buzz. Data are presented normalized to baseline: negative scores thus indicate slowing down and turning less, respectively, while positive scores indicate speeding up and turning more.

All three experiments were performed using buzzes at frequencies of 100 Hz, as in Eschbach et al. (Eschbach et al., 2011), as well as buzzes of one order of magnitude lower and higher frequency (10 Hz, 1000 Hz). All experiments comply with applicable law and regulations.

### Statistics

Statistical analyses were non-parametric throughout and performed with Statistica on a PC (Statsoft 7.0, Tulsa, USA). To compare across multiple groups, we used Kruskal–Wallis tests (KW); Mann–Whitney U-tests (MWU) were used for pairwise comparisons. To test for differences from chance level we used One-Sample Sign-tests (OSS). In cases of multiple comparisons, we applied a Bonferroni correction by dividing 0.05 by the number of comparisons made (presented as P<0.05/3 in cases of e.g. three comparisons); this ensures a within-experiment error rate below 5%. Results of statistical analyses are presented in the figure legends. Data are presented as box-whisker plots (middle line: median; box: lower and upper quartile; whiskers: 90th and 10th percentile).

### List of abbreviations

AM: *n*-amyl acetate; OCT: octanol; TRP: transient receptor potential; NOMPC: No mechanoreceptor potential C; NAN: nanchung; IAV: inactive.

## Supplementary Material

Supplementary Material
