# Supplementary material for: Immediate and punitive impact of mechanosensory disturbance on olfactory behaviour of larval Drosophila
Source: Biol Open. 2014 Sep 26;3(10):1005–10. doi: 10.1242/bio.20149183 (PMC4197435; doi:10.1242/bio.20149183)
Supplement: Supplementary Material [file supp_3_10_1005__index.html]

Immediate and punitive impact of mechanosensory disturbance on olfactory behaviour of larval Drosophila — Immediate and punitive impact of mechanosensory disturbance on olfactory behaviour of larval Drosophila — Supplementary Material 

# Immediate and punitive impact of mechanosensory disturbance on olfactory behaviour of larval *Drosophila*

## bio.20149183 Supplementary Material

**Files in this Data Supplement:**

- Supplementary Material - Timo Saumweber et al. doi: 10.1242/bio.20149183
