# Supplementary material for: Immediate and punitive impact of mechanosensory disturbance on olfactory behaviour of larval Drosophila
Source: Biol Open. 2014 Sep 26;3(10):1005–10. doi: 10.1242/bio.20149183 (PMC4197435; doi:10.1242/bio.20149183)
Supplement: Supplementary Material [file supp_bio.20149183_bio.20149183-s1.pdf]

Biology Open (2014) 000, 1–6 doi:10.1242/bio.20149183

Supplementary Material

Timo Saumweber et al. doi: 10.1242/bio.20149183

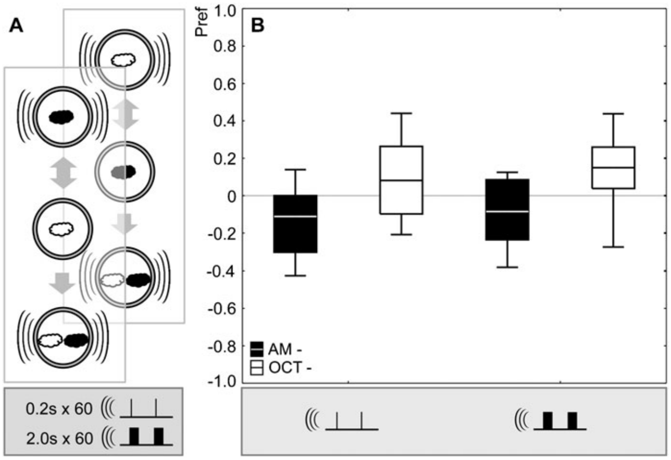

Fig. S1. Buzz as punishment. (A) Experimental design. (B) Plotted are the preference scores of reciprocally trained groups of larvae from the experiment displayed in Fig. 1D. The filled box plots indicate AM preference when AM was punished during training (AM–) whereas the open box plots indicate the AM preference for the reciprocally trained group (OCT–).

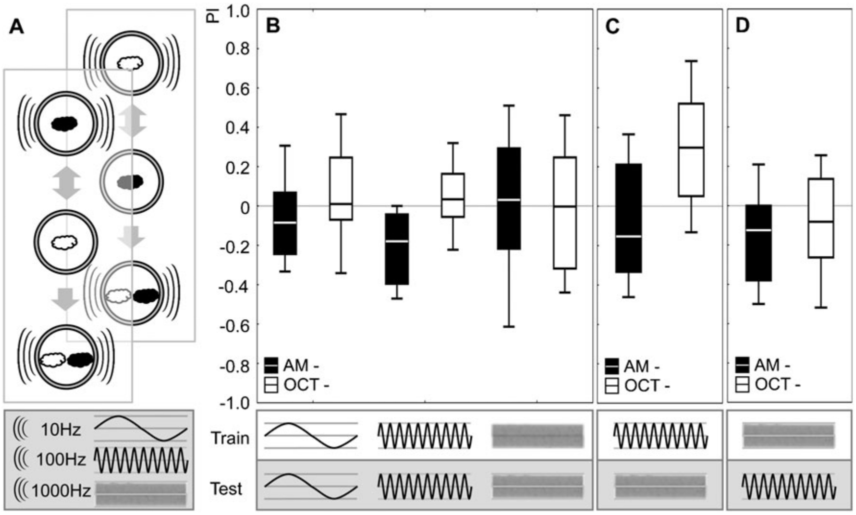

Fig. S2. Buzz as punishment: frequency-dependence. (A) Experimental design. (B–D) Plotted are the preference scores of reciprocally trained groups of larvae from the experiment displayed in Fig. 2. Other details as in supplementary material Fig. S1.

Biology Open (2014) 000, 1–6 doi:10.1242/bio.20149183

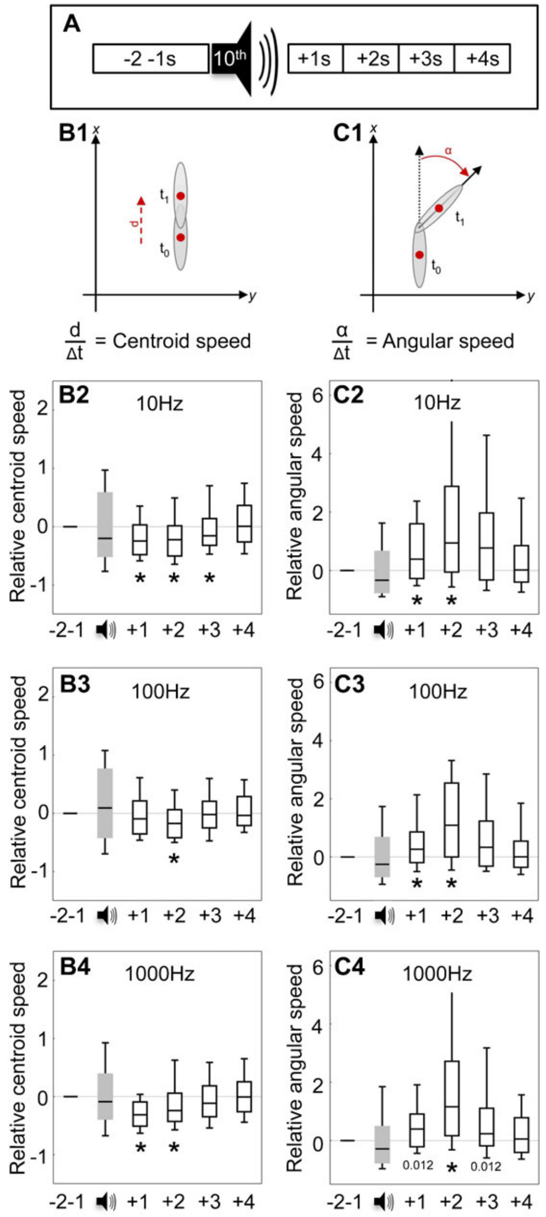

Fig. S3. Buzz as modulator of locomotion after 10th buzz. Same as Fig. 4, for the 10th buzz.

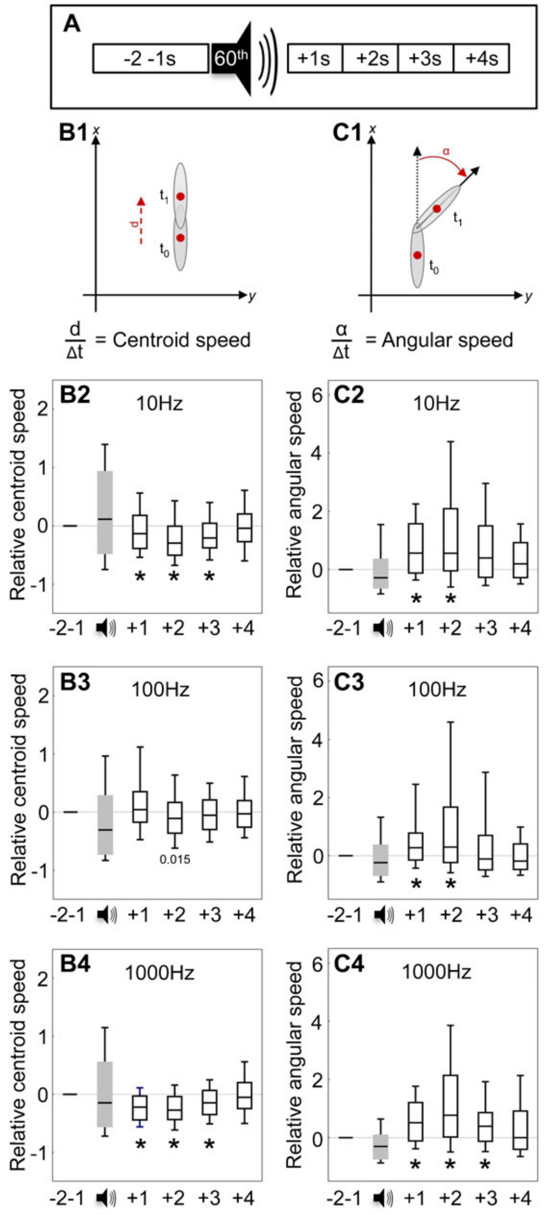

Fig. S4. Buzz as modulator of locomotion after 60th buzz. Same as Fig. 4 and supplementary material Fig. S3, for the 60th buzz.

Biology Open

Biology Open (2014) 000, 1–6 doi:10.1242/bio.20149183

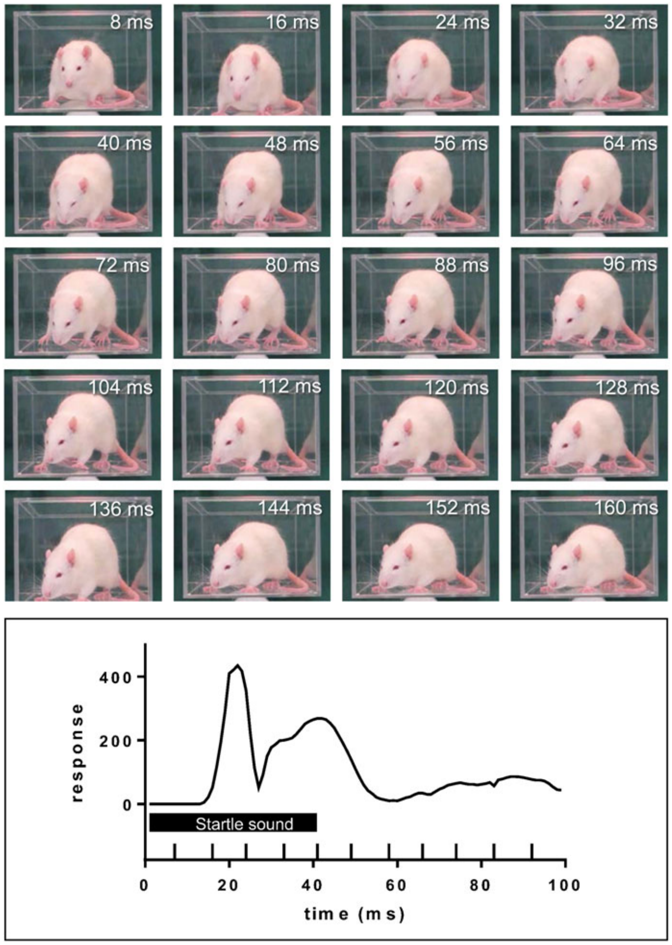

Fig. S5. Acoustic startle of a rat. A startle system (SR-LAB, San Diego Instruments, San Diego, CA) was used that contained a small custom-made enclosure made of a transparent Plexiglas cylinder (12×12×12 cm). Movements of the animals were detected by motion-sensitive transducers mounted underneath. The output signal of the transducers was digitized (sampling rate: 1 kHz) and stored on a PC. The acoustic startle probe (40 ms, 120 dB SPL white noise) was generated by high-frequency loudspeakers mounted in the centre of the ceiling of the test chambers. A male Sprague–Dawley rat was exposed to a startle stimulus of 40 ms duration. The startle response was videotaped by a digital camera (Canon, Powershot SX50) in the slow-motion mode. The figures show that startle behaviour is biphasic (Koch, 1999; Yeomans and Frankland, 1995). The first phase is protective, in particular for the sense organs and the dorsal surface of the neck: the eyes are closed (ca. 16–40 ms), the ears are flattened, the neck is stiffened and the body bent (from ca. 24 ms on), and the legs are straightened (ca. 48 ms). The second phase serves to locate the threat and to prepare a fight or flight decision: the eyes are opened (from ca. 40 ms on), ears pricked (from ca. 96 ms on), and legs lifted. The sketch below indicates a timeline for the presentation of the startle sound (bar) and for the first frames of the picture series shown above. The curve is the voltage output (mV) of the piezoelectric element measuring the startle response.

Biology Open
